# Incidence and Sex-Based Comparison of Prosthesis-Patient Mismatch in Patients Undergoing Transcatheter Aortic Valve Replacement

**DOI:** 10.1016/j.jscai.2025.103621

**Published:** 2025-05-01

**Authors:** Karim Al-Azizi, Taylor Pickering, Mohamad Bader AboHajar, Ghadi Moubarak, Asim Mohiuddin, Srinivasa P. Potluri, Sibi Thomas, Imran Baig, Obadah Aqtash, Sanjeev Trehan, Katherine B. Harrington, Justin M. Schaffer, William T. Brinkman, Amro Alsaid, Zuyue Wang, Jonathan Ladner, Rahul Gunukula, Colleen Parro, Tanushree Prasad, Robert Stoler, Yashasvi Chugh, Subhash Banerjee, Timothy Mixon, Robert J. Widmer, Angel Caldera, Jose Condado Contreras, Anita Krueger, Janaki Rami Reddy Manne, Prajakta Phatak, Ralph M. Matar, William Gray, J. Michael DiMaio, Michael J. Mack, Molly Szerlip

**Affiliations:** a Department of Cardiology, Baylor Scott & White The Heart Hospital, Plano, Texas; b Baylor Scott & White Research Institute, Plano, Texas; c Department of Cardiothoracic Surgery, Baylor Scott & White The Heart Hospital, Plano, Texas; d Department of Cardiology, Baylor University Medical Center, Dallas, Texas; e Department of Cardiology, Baylor Scott & White Medical Center, Temple, Texas; f Department of Cardiology, Baylor Scott & White Medical Center, Round Rock, Texas; g Department of Cardiothoracic Surgery, Baylor Scott & White All Saints Medical Center, Fort Worth, Texas; h Department of Cardiology, Baylor Scott & White Medical Center, College Station, Texas

**Keywords:** balloon-expandable valves, prosthesis-patient mismatch, self-expanding valves, sex differences, transcatheter aortic valve replacement

## Abstract

**Background:**

Prosthesis-patient mismatch (PPM) remains a topic of controversy in patients undergoing transcatheter aortic valve replacement (TAVR), particularly among women with smaller annuli. Although self-expanding valves (SEV) appear to provide superior hemodynamic performance than balloon-expandable valves, the impact of PPM severity on long-term survival, particularly regarding sex-specific differences, remains inadequately characterized.

**Methods:**

This retrospective cohort study analyzed 3016 patients (1338 women) who underwent native valve TAVR from 2012 to 2021. Patients were stratified by sex, valve type (balloon-expandable valve vs SEV), and PPM predicted (PPM_P_) and measured (PPM_M_) severity as defined by the Valve Academic Research Consortium. The primary outcome was all-cause mortality at 5 years, with secondary outcomes including PPM incidence, severity, and residual transvalvular gradients.

**Results:**

Women exhibited higher rates of severe PPM_P_ (1.7% vs 0.1%; *P* < .001) and severe PPM_M_ (7.3% vs 5.4%; *P* = .033). Notably, neither moderate nor severe PPM_P_ or PPM_M_ adversely affected 5-year survival in women (severe PPM_P_ hazard ratio [HR], 1.24; *P* = .709; severe PPM_M_ HR, 1.35; *P* = .168). SEVs were associated with lower overall PPM_P_ (12.8% vs 31.8%) and PPM_M_ (16.1% vs 31.1%) and superior hemodynamics. Although SEVs demonstrated a nonsignificant lower unadjusted survival (44.4% vs 38.0%; *P* = .286), 5-year survival was similar within PPM strata after risk adjustment (overall PPM_P_ HR, 0.51; *P* = .510; overall PPM_M_ HR, 0.77; *P* = .412).

**Conclusions:**

Despite a higher incidence and severity of both PPM_P_ and PPM_M_, women did not experience decreased long-term survival after TAVR. Additionally, there was no risk-adjusted survival difference between valve types, emphasizing the need for individualized prosthesis selection and lifetime valve management considerations.

## Introduction

Prosthesis-patient mismatch (PPM) is defined by an effective orifice area (EOA) that is too small for the patient’s body surface area (BSA), resulting in higher transvalvular gradients and impaired hemodynamics.^1^^,^^2^ Despite advancements in transcatheter aortic valve replacement (TAVR) technologies designed to mitigate PPM risk, its prevalence varies significantly based on valve type, patient demographics, and procedural factors.^3^, ^4^, ^5^, ^6^, ^7^, ^8^ Among these factors, sex-related disparities in TAVR outcomes have garnered increasing attention. Women undergoing TAVR exhibit unique anatomical and physiological characteristics, such as smaller annular dimensions and higher prevalence of small valve sizes, predisposing them to higher rates of PPM.^5^^,^^9^, ^10^, ^11^, ^12^ However, these characteristics do not uniformly translate into poorer outcomes. Women often demonstrate paradoxically similar survival compared to men, even in the presence of PPM, suggesting that there are sex-specific mechanisms that influence tolerance to PPM.^8^, ^9^, ^10^, ^11^^,^^13^

Although previous studies have demonstrated that self-expanding valves (SEV) are associated with superior hemodynamics due to larger EOA than balloon-expandable valves (BEV), the relationship between valve type, PPM severity, and sex-specific outcomes remains incompletely understood.^4^, ^5^, ^6^^,^^14^ Furthermore, while moderate and severe PPM have been linked to adverse outcomes, including impaired regression of left ventricular hypertrophy, increased rehospitalization, and reduced survival in the surgical literature, the extent to which these risks differ by sex has not been adequately characterized in patients undergoing TAVR.^4^^,^^7^^,^^11^ This study aimed to evaluate sex-specific differences in PPM incidence, severity, and outcomes among patients undergoing TAVR. Specifically, we assessed the long-term survival outcomes of women and men with predicted PPM (PPM_P_) and measured PPM (PPM_M_), examined the role of valve type (BEV vs SEV) in mitigating PPM, and analyzed the interaction between PPM severity and sex and valve type on 1- and 5-year mortality.

## Methods

This study was a retrospective analysis of patients who underwent TAVR between 2012 and 2021 at Baylor Scott & White Health Care System. The study included patients who received commercially available BEV (SAPIEN, SAPIEN XT, SAPIEN 3; Edwards Lifesciences) or SEV (CoreValve, Evolut R, Evolut Pro; Medtronic). Patients that received a valve-in-valve TAVR were excluded, as were those with missing postoperative EOA. Ultimately, 3016 patients with complete clinical, procedural, and echocardiographic follow-up data were analyzed. Patients were categorized based on sex (male vs female) and further stratified by valve type (BEV vs SEV). Subgroups were further defined by the severity of PPM, classified as PPM_P_ and PPM_M_.

PPM was defined according to the Valve Academic Research Consortium recommendations, consistent with prior studies evaluating PPM in TAVR patients.^15^ Complete clinical, procedural, and echocardiographic follow-up data were analyzed to assess outcomes. The predicted EOA indexed to BSA (EOAi) was calculated using published EOA reference values for each type and size of transcatheter bioprosthesis (Supplemental Table S1) divided by the patient’s BSA.^16^ EOAi thresholds for severity were categorized as none if EOAi was >0.85 cm^2^/m^2^, moderate if EOAi ranged from 0.65 to 0.85 cm^2^/m^2^, and severe if EOAi was <0.65 cm^2^/m^2^. Additionally, these values were adjusted for patients with a body mass index of ≥30 kg/m^2^ as follows: none if EOAi >0.70 cm^2^/m^2^, moderate if EOAi >0.55 and ≤0.70 cm^2^/m^2^, and severe if EOAi ≤0.55 cm^2^/m^2^.

Patient demographics, clinical comorbidities, and procedural characteristics were retrospectively obtained from the electronic medical record. The primary outcome was all-cause mortality at 5 years after TAVR. Secondary outcomes included the incidence and severity of PPM_P_ and PPM_M_, postprocedural residual gradients (mean gradient ≥20 mm Hg), and 30-day and 1-year survival, stratified by sex and valve type. This study was approved by our institutional review board and ethics committee. Given the retrospective nature of the study, patient informed consent was waived.

Descriptive statistics were used to summarize baseline demographics, procedural characteristics, and outcomes. Continuous variables are presented as medians with IQR and categorical variables as counts and percentages. Differences between groups were assessed using the Mann–Whitney U test for continuous variables and chi-square or Fisher exact tests for categorical variables. Survival analyses were performed using Kaplan–Meier curves with log-rank testing to compare survival between groups. Cox proportional hazards regression models were constructed to evaluate the impact of PPM severity, valve type, and sex on survival, adjusting for covariates such as age, Society of Thoracic Surgeons risk score, valve size, and baseline hemodynamic parameters. Interaction terms for sex, valve type, and PPM severity were included to assess differential effects. Adjusted hazard ratios (HR) with 95% CI were reported. Statistical significance was defined as *P* < .05.

## Results

### Baseline characteristics

A total of 3016 patients who underwent native valve TAVR were included in this study, of whom 1338 (44.4%) were female and 1678 (55.6%) were male. Women were significantly older than men (80.7 vs 79.7 years, *P* = .006) and had a smaller BSA (1.81 vs 2.06 m^2^, *P* < .001) on average despite a higher rate of obesity (body mass index ≥30 kg/m^2^; 39.2% vs 35.3%, *P* = .028). Women also exhibited higher Society of Thoracic Surgeons risk scores than men (5.5 vs 4.2, *P* < .001) despite having lower rates of prior myocardial infarction (16.1% vs 21.9%, *P* < .001), prior percutaneous coronary intervention (34.0% vs 46.9%, *P* < .001), prior coronary artery bypass grafting (12.1% vs 30.0%, *P* < .001), peripheral artery disease (18.1% vs 24.6%, *P* < .001), and smoking history (9.5% vs 14.2%, *P* < .001; Table 1).

**Table 1:** Demographic and baseline echocardiographic characteristics by sex and valve type (BEV vs SEV).

	All patients (N = 3016)	Women (n = 1338)	Men (n = 1678)	*P*	BEV (n = 946)	SEV (n = 392)	*P*
Baseline clinical and demographics							
Age, y	80.1 (73.3-85.6)	80.7 (73.4-86.0)	79.7 (73.2-85.2)	.0055	80.5 (72.8-85.3)	81.2 (74.7-86.4)	.1181
Race							
White	2831 (93.9)	1236 (92.4)	1595 (95.0)	.0023	878 (92.8)	358 (91.3)	.3514
Black	142 (4.7)	79 (5.9)	63 (3.8)	.0056	48 (5.1)	31 (7.9)	.0453
Asian	37 (1.2)	20 (1.5)	17 (1.0)	.2325	17 (1.8)	3 (0.8)	.1569
BMI, kg/m^2^	27.9 (24.3-32.4)	28.1 (23.8-33.0)	27.7 (24.5-32.0)	.6446	28.2 (23.8-33.2)	27.9 (24.0-32.5)	.4006
BMI ≥30 kg/m^2^	1116 (37.0)	524 (39.2)	592 (35.3)	.0282	384 (40.6)	140 (35.7)	.0962
BSA, m^2^	1.96 (1.77-2.15)	1.81 (1.64-2.00)	2.06 (1.91-2.24)	<.001	1.81 (1.64-2.01)	1.80 (1.64-1.97)	.3003
STS risk score	4.7 (2.7-7.6)	5.5 (3.3-8.5)	4.2 (2.4-6.8)	<.001	5.2 (3.1-7.9)	6.5 (4.0-9.5)	<.001
Hypertension	2693 (89.3)	1197 (89.5)	1496 (89.2)	.7857	846 (89.4)	351 (89.5)	.9517
Diabetes	1226 (40.7)	544 (40.7)	682 (40.6)	.9938	394 (41.7)	150 (38.3)	.2514
Current dialysis	84 (2.9)	38 (2.9)	46 (2.8)	.8836	30 (3.3)	8 (2.1)	.2333
Prior MI	470 (19.3)	174 (16.1)	296 (21.9)	.0003	121 (16.3)	53 (15.6)	.7582
AF or flutter	1082 (35.9)	458 (34.2)	624 (37.2)	.0926	323 (34.1)	135 (34.4)	.9176
Pacemaker	439 (14.6)	181 (13.5)	258 (15.4)	.1528	123 (13.0)	58 (14.8)	.3826
Prior PCI	1242 (41.2)	455 (34.0)	787 (46.9)	<.001	320 (33.8)	135 (34.4)	.8297
Prior CABG	666 (22.1)	162 (12.1)	504 (30.0)	<.001	121 (12.8)	41 (10.5)	.2341
Previous AV repair	1 (0.03)	1 (0.1)	0 (0.0)	.4436	0 (0.0)	1 (0.3)	.293
Previous AV balloon	210 (7.0)	98 (7.3)	112 (6.7)	.4861	80 (8.5)	18 (4.6)	.0135
Creatinine, mg/dL	1.14 (0.94-1.44)	1.04 (0.85-1.33)	1.21 (1.01-1.51)	<.001	1.04 (0.85-1.33)	1.04 (0.84-1.32)	.7767
Prior PAD	654 (21.7)	242 (18.1)	412 (24.6)	<.001	172 (18.2)	70 (17.9)	.8883
Smoker	365 (12.1)	127 (9.5)	238 (14.2)	<.001	101 (10.7)	26 (6.6)	.0216
Baseline echocardiography							
Mean aortic valve gradient, mm Hg	43.0 (38.0-51.0)	44.0 (39.0-51.5)	42.0 (37.0-50.0)	<.001	45.0 (39.0-52.0)	42.0 (38.0-51.0)	.0171
Mean aortic valve area, cm^2^	0.70 (0.60-0.80)	0.70 (0.50-0.80)	0.70 (0.60-0.85)	<.001	0.67 (0.50-0.80)	0.70 (0.54-0.80)	.0583
Aortic regurgitation (≥ moderate)	382 (12.7)	179 (13.4)	203 (12.1)	.2935	117 (12.4)	62 (15.8)	.0917

Values are presented as median (IQR) for continuous variables and as count (percentage) for categorical variables. *P* values represent comparisons between groups, as indicated.

AF, atrial fibrillation; AV, aortic valve; BEV, balloon-expandable valve; BMI, body mass index; BSA, body surface area; CABG, coronary artery bypass grafting; MI, myocardial infarction; PAD, peripheral artery disease; PCI, percutaneous coronary intervention; SEV, self-expanding valve; STS, Society of Thoracic Surgeons.

### Echocardiographic parameters and PPM

As described in Table 1, baseline echocardiographic data demonstrated high mean aortic valve gradients (44.0 mm Hg vs 42.0 mm Hg, *P* < .001) between women and men, respectively. Procedural characteristics revealed that SEV were more frequently implanted in women than men (29.3% vs 21.7%; *P* < .001), and women were more likely to receive smaller valves (23 mm vs 26 mm; *P* < .001). Postprocedural echocardiographic data demonstrated higher mean prosthetic gradients in women than in men (9.0 vs 8.0 mm Hg; *P* < .001; Supplemental Figure S1) as well as a higher rate of residual gradients ≥20 mm Hg (5.2% vs 1.9%; *P* < .001). The overall prevalence of PPM was greater in women than in men, irrespective of the method used to define PPM. PPM_P_ occurred in 26.2% of women vs 18.2% of men (*P* < .001), while PPM_M_ was observed in 26.7% of women compared to 24.1% of men (*P* = .111; Table 2). Severe PPM rates also differed significantly by sex, with PPM_P_ at 1.7% in women vs 0.1% in men (*P* < .001) and PPM_M_ at 7.3% in women compared to 5.4% in men (*P* = .033).

**Table 2:** Procedural, baseline, and postoperative echocardiographic characteristics by sex and valve type (BEV vs SEV).

	All patients (N = 3016)	Women (n = 1338)	Men (n = 1678)	*P*	BEV (n = 946)	SEV (n = 392)	*P*
Procedural characteristics							
Urgent/emergent procedure	95 (6.3)	42 (6.3)	53 (6.3)	.9724	30 (7.8)	12 (4.3)	.0724
Transfemoral approach	2874 (95.3)	1268 (94.8)	1606 (95.7)	.2255	884 (93.5)	384 (98.0)	.0007
Valve type							
BEV	2260 (74.9)	946 (70.7)	1314 (78.3)	<.001	–	–	
SEV	756 (25.1)	392 (29.3)	364 (21.7)	<.001	–	–	
Valve size, mm	26.0 (23.0-29.0)	23.0 (23.0-26.0)	26.0 (26.0-29.0)	<.001	23.0 (23.0-26.0)	26.0 (26.0-29.0)	<.001
Small valves (BEV ≤23 mm, SEV ≤26 mm)	1074 (35.6)	924 (69.1)	150 (8.9)	<.001	661 (69.9)	263 (67.1)	.3165
Postoperative echocardiography							
LVEF, %	60.0 (54.0-65.0)	63.0 (59.0-67.5)	60.0 (50.0-65.0)	<.001	62.7 (60.0-65.5)	63.0 (57.6-69.0)	.8271
Prosthetic mean gradient, mm Hg	9.0 (6.0-12.0)	9.0 (6.0-13.0)	8.0 (6.0-11.0)	<.001	10 (8-14)	6 (4-9)	<.001
High residual gradient (mean gradient ≥ 20 mm Hg)	102 (3.4)	70 (5.2)	32 (1.9)	<.001	63 (6.7)	7 (1.8)	.0003
Measured EOA, cm^2^/m^2^	1.90 (1.55-2.30)	1.70 (1.40-2.10)	2.0 (1.7-2.4)	<.001	1.6 (1.4-2.0)	1.9 (1.6-2.3)	<.001
Measured EOAi, cm^2^/m^2^	0.96 (0.79-1.17)	0.94 (0.77-1.16)	0.97 (0.80-1.19)	.0073	0.9 (0.7-1.1)	1.1 (0.9-1.3)	<.001
Moderate or severe aortic regurgitation	94 (3.1)	50 (3.7)	44 (2.6)	.0801	33 (3.5)	17 (4.3)	.4565
SVI, mL/m^2^	36.6 (29.5-45.3)	37.7 (30.0-45.6)	35.6 (29.2-44.9)	.0047	38.8 (31.0-46.4)	35.0 (28.5-43.8)	.0001
SVI ≤35 mL/m^2^	948 (12.7)	385 (41.1)	563 (48.1)	.0013	253 (37.6)	132 (50.0)	.0005
Predicted PPM							
Predicted EOAi, cm^2^/m^2^	0.88 (0.79-0.98)	0.88 (0.79-0.98)	0.88 (0.80-0.98)	.0063	0.84 (0.76-0.93)	0.98 (0.88-1.08)	<.001
Overall predicted PPM	657 (21.8)	351 (26.2)	306 (18.2)	<.001	301 (31.8)	50 (12.8)	<.001
Predicted PPM severity							
None	2359 (78.2)	987 (73.8)	1372 (81.7)	<.001	645 (68.2)	342 (87.2)	<.001
Moderate	633 (21.0)	328 (24.5)	305 (18.2)	<.001	296 (31.3)	32 (8.2)	<.001
Severe	24 (0.8)	23 (1.7)	1 (0.1)	<.001	5 (0.5)	18 (4.6)	<.001
Measured PPM							
Measured EOAi, cm^2^/m^2^	0.96 (0.79-1.17)	0.94 (0.77-1.16)	0.97 (0.80-1.19)	.0073	0.9 (0.7-1.1)	1.1 (0.9-1.3)	<.01
Overall measured PPM	762 (25.3)	357 (26.7)	405 (24.1)	.1109	294 (31.1)	63 (16.1)	<.001
Measured PPM severity							
None	2249 (74.7)	979 (73.3)	1270 (75.8)	.1109	651 (68.9)	328 (83.9)	<.001
Moderate	573 (19.0)	259 (19.4)	314 (18.8)	.6567	216 (22.9)	43 (11.0)	<.001
Severe	189 (6.3)	98 (7.3)	91 (5.4)	.0325	78 (8.3)	20 (5.1)	.0453
Overall mortality	1248 (41.4)	533 (39.8)	715 (42.6)	.1243	359 (38.0)	174 (44.4)	.0286

Values are presented as median (IQR) for continuous variables and as count (percentage) for categorical variables. *P* values represent comparisons between groups, as indicated. Moderate PPM was defined as an EOAi between 0.65 and 0.85 cm^2^/m^2^ and severe PPM as an EOAi <0.65 cm^2^/m^2^. Adjusted EOAi thresholds for patients with BMI ≥30 kg/m^2^ were used as follows: moderate PPM (0.55-0.70 cm^2^/m^2^), severe PPM (≤0.55 cm^2^/m^2^).

BEV, balloon-expandable valve; EOA, effective orifice area; EOAi, indexed effective orifice area; LVEF, left ventricular ejection fraction; PPM, prosthesis-patient mismatch; SEV, self-expanding valve; SVI, stroke volume index.

### Valve type comparisons in female patients

Women who received a BEV had significantly higher echocardiographic baseline mean aortic valve gradients than those receiving an SEV (45.0 mm Hg vs 42.0 mm Hg; *P* = .017) despite having similar aortic valve areas (Table 1, Supplemental Figure S2). Post procedure, BEV were associated with higher transvalvular gradients (10 mm Hg vs 6 mm Hg; *P* < .001; Table 2) and a greater incidence of residual gradients ≥20 mm Hg (6.7% vs 1.8%; *P* < .001) compared to SEV. Additionally, PPM prevalence was significantly higher among BEV recipients, with PPM_P_ present in 31.8% vs 12.8% in SEV recipients (*P* < .001) and PPM_M_ in 31.1% vs 16.1% (*P* < .001). Notably, severe PPM_P_ was less frequent in BEV recipients (0.5% vs 4.6%; *P* < .001), whereas severe PPM_M_ was more common in BEV recipients (8.3% vs 5.1%; *P* = .045).

### Effect of PPM on survival outcomes by sex

**Figure 1:**
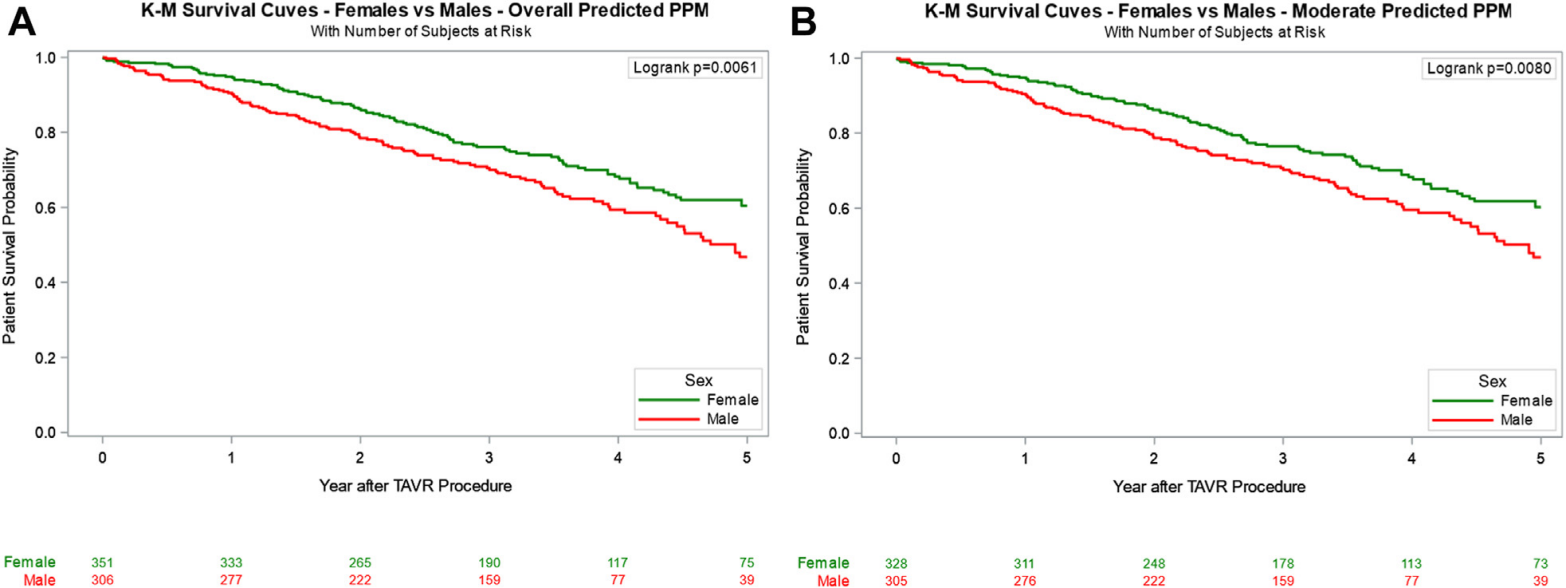

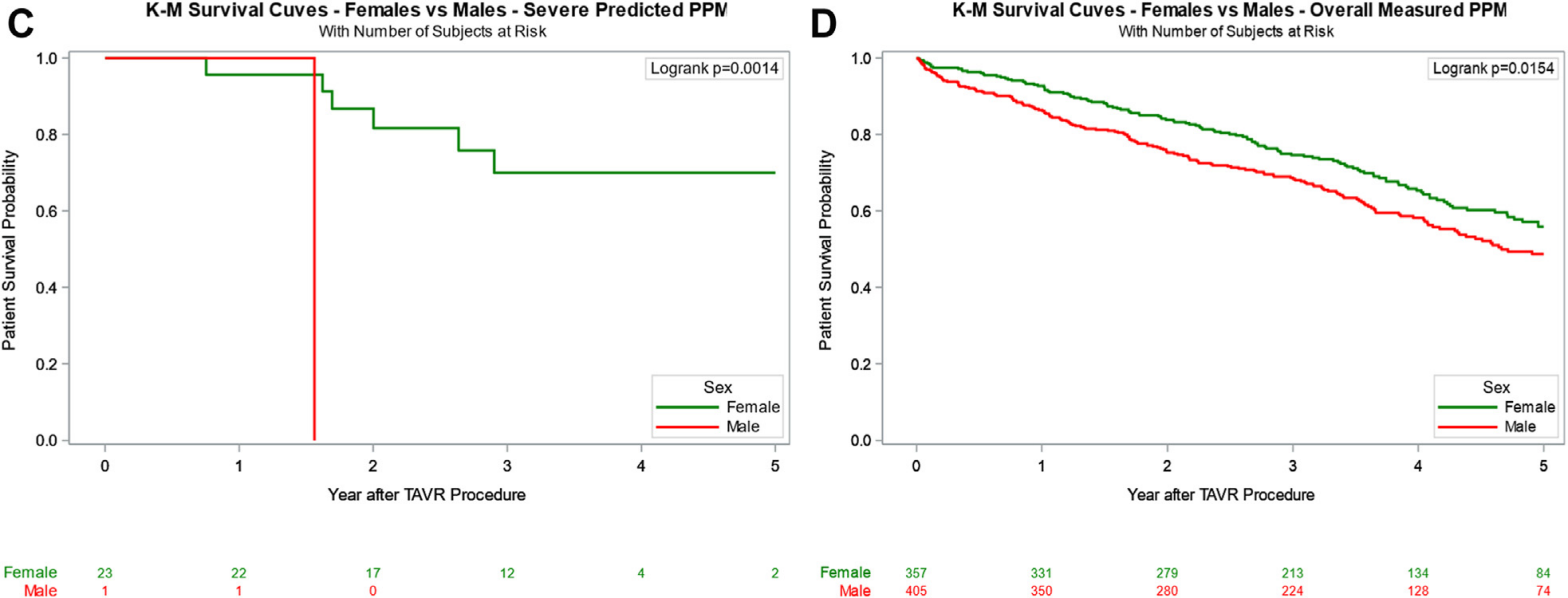

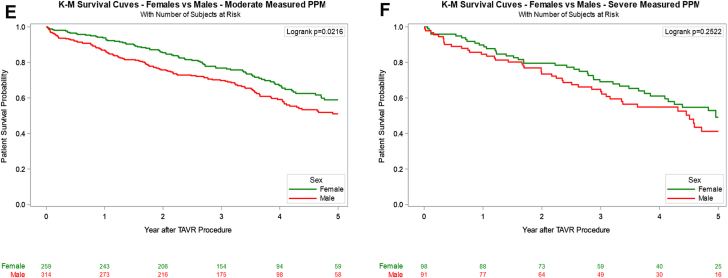
**Five-year Kaplan–Meier (K-M) survival analysis stratified by sex and predicted or measured prosthesis-patient mismatch severity.** (A) Overall PPM_P_. (B) Moderate PPM_P_. (C) Severe PPM_P_. (D) Overall PPM_M_. (E) Moderate PPM_M_. (F) Severe PPM_M_. PPM_M_, measured prosthesis-patient mismatch; PPM_P_, predicted prosthesis-patient mismatch; TAVR, transcatheter aortic valve replacement.

Overall survival was not significantly different between women and men (39.8% vs 42.6%, *P* = .124) at a median follow-up of 40.2 (IQR, 23.1-54.5) months. Upon further Kaplan–Meier log-rank analysis stratified by PPM severity, women demonstrated consistently improved survival compared to men in all categories except severe PPM_M_ (Figure 1). In women, however, neither moderate nor severe PPM_P_ significantly impacted 5-year survival, with an adjusted HR of 1.24 (95% CI, 0.41-3.75; *P* = .709) for severe PPM_P_ compared to no PPM_P_ (Table 3, Central Illustration). Similar findings were seen with PPM_M_, such that severe PPM_M_ had an adjusted HR of 1.35 (95% CI, 0.88-2.08; *P* = .168). In contrast, men exhibited a survival disadvantage with increasing PPM_P_ severity, although this trend was not observed for PPM_M_ (Supplemental Figure S3). Moderate PPM_P_ in men was associated with a significantly lower 5-year survival rate, with an adjusted HR of 1.51 (95% CI, 1.11-2.06; *P* = .010). Only 1 male patient had severe PPM_P_ with an adjusted HR of 7.25 (95% CI, 0.95-55.05; *P* = .056). Severe PPM_M_ did not significantly affect long-term survival, with an adjusted HR of 1.44 (95% CI, 0.97-2.14; *P* = .070).

**Table 3:** Survival rates in women and men according to predicted and measured PPM.

PPM	Sex	PPM level	30-d survival rate	1-y survival rate	5-y survival rate	HR _Adjusted_	*P*_Adjusted_
PPM_P_	Women	None	98% (97%, 99%)	90% (88%, 91%)	54% (50%, 58%)	Reference	
		Moderate	99% (97%, 100%)	95% (92%, 97%)	60% (53%, 67%)	1.02 (0.71, 1.48)	.9069
		Severe	100% (100%, 100%)	96% (73%, 99%)	70% (44%, 86%)	1.24 (0.41, 3.75)	.7092
		Overall PPM	99% (97%, 100%)	95% (92%, 97%)	60% (54%, 67%)	1.04 (0.73, 1.50)	.8157
	Men	None	99% (98%, 99%)	88% (87%, 90%)	51% (48%, 54%)	Reference	
		Moderate	100% (98%, 100%)	90% (87%, 93%)	47% (39%, 55%)	1.51 (1.11, 2.06)	.0096
		Severe	100% (100%, 100%)	100% (100%, 100%)	0%	7.25 (0.95, 55.05)	.0557
		Overall PPM	100% (98%, 100%)	90% (87%, 93%)	47% (39%, 54%)	1.53 (1.21, 2.09)	.0073
PPM_M_	Women	None	98% (97%, 99%)	90% (88%, 92%)	56% (51%, 59%)	Reference	
		Moderate	99% (97%, 100%)	94% (90%, 96%)	59% (51%, 66%)	1.19 (0.88, 1.63)	.2653
		Severe	99% (93%, 100%)	90% (82%, 94%)	49% (37%, 60%)	1.35 (0.88, 2.08)	.1677
		Overall PPM	99% (97%, 100%)	93% (90%, 95%)	56% (49%, 62%)	1.24 (0.93, 1.64)	.1392
	Men	None	99% (99%, 100%)	90% (88%, 91%)	51% (48%, 55%)	Reference	
		Moderate	97% (94%, 98%)	87% (83%, 90%)	51% (44%, 58%)	1.09 (0.95, 1.42)	.504
		Severe	98% (92%, 99%)	85% (75%, 91%)	41% (29%, 53%)	1.44 (0.97, 2.14)	.0695
		Overall PPM	97% (95%, 98%)	86% (83%, 89%)	49% (43%, 55%)	1.16 (0.92, 1.48)	.2162

Values are presented as percentages with 95% CI. Survival rates were estimated using Kaplan–Meier analysis, with HR adjusted for age, Society of Thoracic Surgeons risk score, valve size, and baseline hemodynamic parameters. *P* values represent comparisons within each sex group using Cox proportional hazards regression analysis.

HR, hazard ratio; PPM, prosthesis-patient mismatch; PPM_M_, measured prosthesis-patient mismatch; PPM_P_, predicted prosthesis-patient mismatch.

**Central Illustration:**
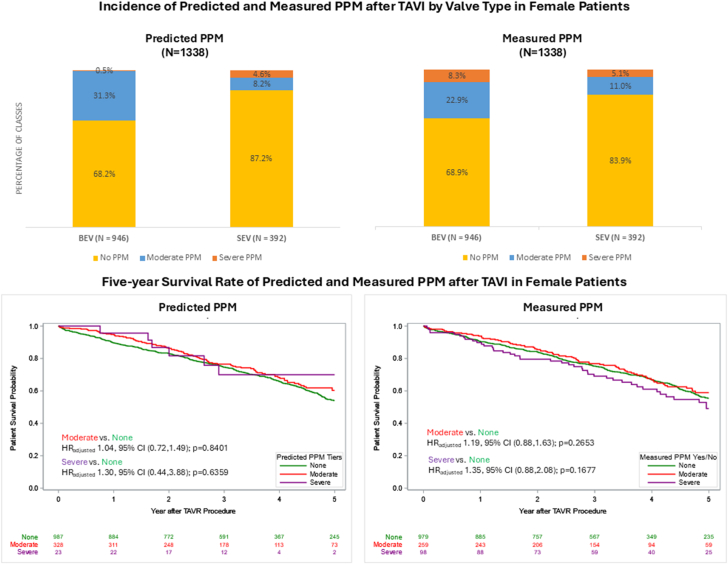
**Incidence andlong-termsurvival of predicted PPM and measured PPM after TAVR in female patients.** BEV, balloon-expandable valve; HR, hazard ratio; PPM, prosthesis-patient mismatch; SEV, self-expanding valve; TAVI, transcatheter aortic valve implantation.

### Valve type and survival in female patients

**Figure 2:**
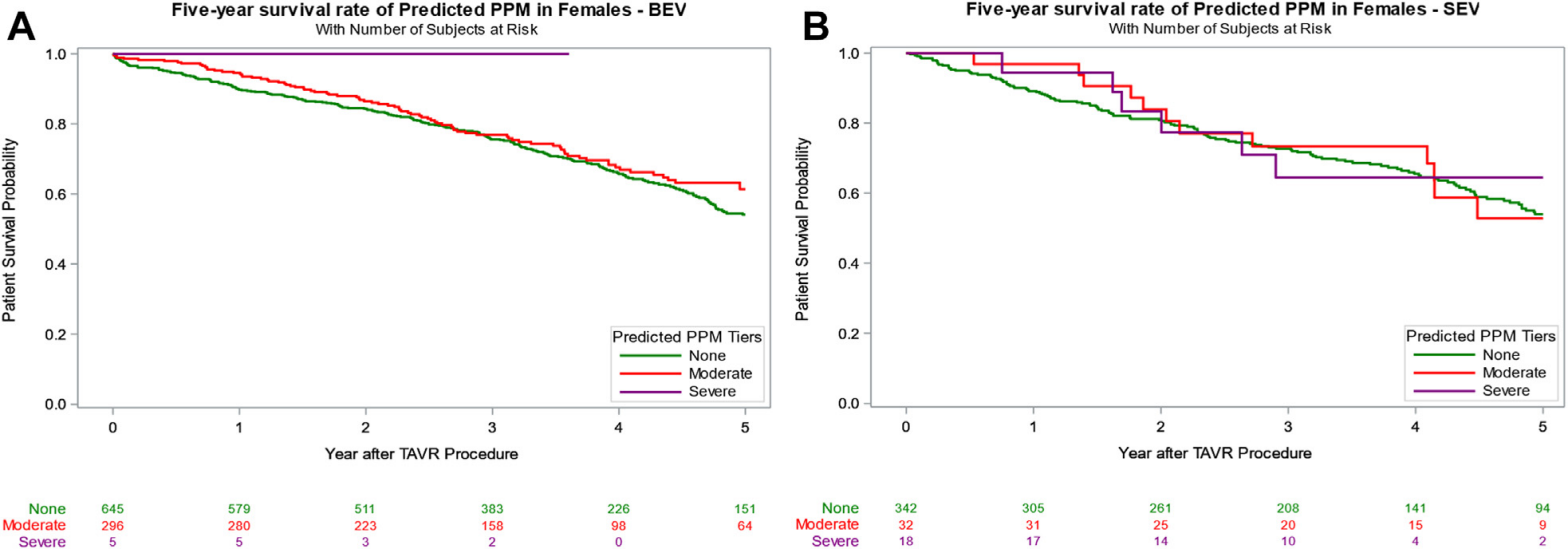

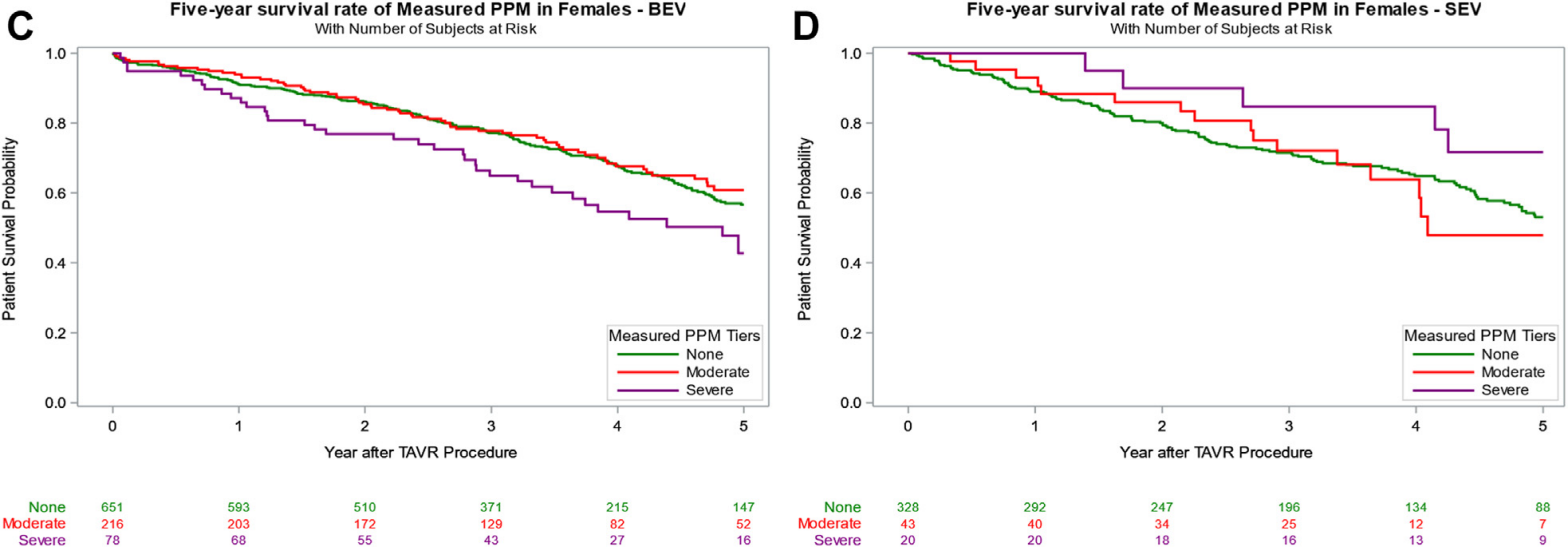
**Kaplan–Meier 5-year survival analysis with adjusted hazard ratios comparing moderate and severe predicted PPM and measured PPM to no PPM in female patients receiving either BEV or SEV.** BEV, balloon-expandable valve; PPM, prosthesis-patient mismatch; SEV, self-expanding valve; TAVR, transcatheter aortic valve replacement.

Overall unadjusted survival among female patients favored SEV (44.4%) over BEV (38.0%), *P* = .029. Kaplan–Meier log-rank analyses stratified by PPM_P_ and PPM_M_ severity were performed to assess survival differences by valve type. This analysis failed to demonstrate a significant survival difference between BEV and SEV recipients across PPM severity, with the exception of severe PPM_M_, which favored SEV (Supplemental Figure S4). However, after risk adjustment, 5-year survival remained similar across PPM severity. Among female patients without PPM_P_, 5-year adjusted survival was similar between those receiving BEV and those receiving SEV (54% vs 54%; *P* = .933; Table 4 and Figure 2). In the moderate PPM_P_ subgroup, BEV recipients demonstrated a numerically higher 5-year adjusted survival rate (61% vs 53%), although this difference was not statistically significant (*P* = .349). Severe PPM_P_ was observed exclusively in SEV recipients, who demonstrated a 5-year survival rate of 64% (95% CI, 37%-82%). Similarly, there was no difference in the 5-year adjusted survival of patients who received BEV compared to those receiving SEV without PPM_M_ (57% vs 53%; *P* = .527). Interestingly, the 5-year survival rate between BEV and SEV recipients was significantly different for those with moderate PPM_M_ (61% vs 48%, *P* = .023), in favor of BEV, but not for those with severe PPM_M_ (43% vs 72%; *P* = .212).

**Table 4:** Survival rates in BEV vs SEV in female patients according to predicted and measured PPM.

PPM	PPM level	Valve type	30-d survival rate	1-y survival rate	5-y survival rate	HR _Adjusted_	*P*_Adjusted_
PPM_P_	None	BEV	98% (96%, 99%)	90% (87%, 92%)	54% (49%, 59%)	0.98 (0.67, 1.45)	.9333
		SEV	99% (98%, 100%)	89% (85%, 92%)	54% (48%, 60%)	Reference	
	Moderate	BEV	99% (96%, 100%)	95% (91%, 97%)	61% (54%, 68%)	0.54 (0.15, 1.96)	.3493
		SEV	100% (100%, 100%)	100% (100%, 100%)	53% (31%, 71%)	Reference	
	Severe	BEV	100% (100%, 100%)	NA	NA	0 (0, Inf)	1.0000
		SEV	100% (100%, 100%)	94% (67%, 99%)	64% (37%, 82%)		
	Overall PPM	BEV	99% (97%, 100%)	95% (91%, 97%)	62% (54%, 68%)	0.51 (0.15, 1.77)	.51
		SEV	100% (100%, 100%)	96% (85%, 99%)	54% (35%, 69%)	Reference	
PPM_M_	No PPM	BEV	98% (96%, 99%)	91% (89%, 93%)	57% (52%, 61%)	1.13 (0.77, 1.65)	.5273
		SEV	99% (98%, 100%)	89% (85%, 92%)	53% (46%, 59%)	Reference	
	Moderate	BEV	99% (96%, 100%)	94% (90%, 96%)	61% (52%, 68%)	0.41 (0.19, 0.89)	.0234
		SEV	100% (100%, 100%)	93% (80%, 98%)	48% (27%, 66%)	Reference	
	Severe	BEV	99% (91%, 100%)	87% (78%, 93%)	43% (29%, 55%)	2.38 (0.61, 9.23)	.2117
		SEV	100% (100%, 100%)	100% (100%, 100%)	72% (44%, 87%)	Reference	
	Overall PPM	BEV	99% (96%, 100%)	92% (89%, 95%)	56% (48%, 62%)	0.77 (0.41, 1.44)	.4118
		SEV	100% (100%, 100%)	95% (86%, 98%)	57% (41%, 70%)	Reference	

Values are presented as percentages with 95% CI. Survival rates were estimated using Kaplan–Meier analysis, with HR adjusted for age, Society of Thoracic Surgeons risk score, valve size, and baseline hemodynamic parameters. *P* values represent comparisons within each sex group using Cox proportional hazards regression analysis.

BEV, balloon-expandable valve; HR, hazard ratio; PPM, prosthesis-patient mismatch; PPM_M_, measured prosthesis-patient mismatch; PPM_P_, predicted prosthesis-patient mismatch; SEV, self-expanding valve.

## Discussion

The management of patients with severe aortic stenosis and a small aortic annulus remains a critical area of investigation given concerns for increased risk associated with PPM and impaired valve hemodynamics in this population. The SMART trial, a landmark randomized study by Herrmann et al,^13^ evaluated clinical outcomes and bioprosthetic valve performance in 716 patients with small annuli who underwent TAVR with either a supraannular SEV or a BEV. The study demonstrated noninferiority of the SEV in terms of major adverse clinical outcomes at 12 months while highlighting its superiority in reducing bioprosthetic valve dysfunction, mean gradients, and PPM incidence. Our study builds upon these findings by analyzing a similar patient cohort with a higher overall sample size and an extended follow-up period of 5 years, allowing for a more comprehensive assessment of long-term survival.

In this large, retrospective analysis, multiple key findings emerged regarding the interplay of PPM, sex-specific outcomes, and valve type selection in patients undergoing TAVR. First, we found that women experience higher rates of both PPM_P_ and PPM_M_ than men. Second, women with similar PPM severity demonstrated improved long-term survival than men. Third, there was no difference in long-term survival with increased PPM_P_ or PPM_M_ severity. Fourth, despite the superior hemodynamic profile of the SEV as compared to BEV, overall mortality was higher for patients with SEV. Finally, risk-adjusted survival between SEV and BEV stratified by PPM severity demonstrated no difference.

Consistent with previous research, our study underscores that women, who typically have smaller annuli and BSA, are predisposed to receiving smaller prostheses and thus are more likely to experience higher rates of PPM.^7^^,^^10^^,^^17^, ^18^, ^19^, ^20^, ^21^ Despite higher rates of overall and severe PPM, our analysis demonstrated a paradoxically neutral effect of PPM on women’s long-term survival, aligning with recent data from Al-Azizi et al^3^ and the findings of the SMART trial.^13^ Echoing these studies, we demonstrated that long-term survival was not significantly impacted, even in the presence of severe PPM_P_ or PPM_M_. This finding challenges the current dogma that increasing PPM severity, particularly in women and those with small annuli, portends a poor prognosis.^7^^,^^8^^,^^22^^,^^23^ Several physiological and anatomical differences unique to women may account for this observed difference. Women generally have smaller left ventricular cavities and lower stroke volumes, which may mitigate the hemodynamic burden imposed by higher residual gradients and reduced EOA.^9^^,^^18^ Furthermore, women are known to exhibit superior left ventricular remodeling and improved ventricular compliance after TAVR, which may enhance their ability to adapt to and tolerate PPM.^1^^,^^20^^,^^24^

Our comparison of BEV and SEV in female patients showed that SEV yielded significantly lower postprocedural gradients and lower rates of PPM. These findings are consistent with earlier reports highlighting the superior hemodynamic profiles of SEV, especially in patients with small aortic annuli.^3^^,^^6^^,^^10^^,^^13^ Despite these hemodynamic advantages, overall mortality was higher in patients receiving SEV than in those receiving BEV. As this represents an unmatched patient population, this survival difference may be explained by differences in baseline patient characteristics, procedural nuances, and/or various confounding factors that disproportionately affected SEV recipients. Alternatively, the favorable hemodynamics of SEV may not directly translate into survival benefits due to complex interactions between valve function and patient comorbidities, particularly in high-risk populations. Interestingly, an unadjusted risk subgroup analysis revealed a potential survival advantage for SEV recipients with severe PPM_M_, with a significant difference in survival noted (*P* = .004 by log-rank testing). However, when this difference was further evaluated using a risk-adjusted analysis, there was no increased risk of mortality at 5 years for female patients receiving either BEV or SEV, regardless of PPM_P_ or PPM_M_ severity.^3^^,^^13^^,^^25^ This finding aligns with those of the SMART trial,^13^ which demonstrated that while SEV recipients had lower mean gradients and a reduced incidence of severe PPM at 1 year, this hemodynamic advantage did not translate into a clear survival benefit after risk adjustment. Similarly, long-term follow-up from the CHOICE trial^25^ revealed that despite the superior forward-flow hemodynamics associated with SEV, there was no statistically significant difference in all-cause mortality at 5 years between BEV and SEV recipients (53.4% vs 47.6%, *P* = .38).

Considering these findings, survival after TAVR is dependent on multiple clinical and anatomical factors, suggesting that prosthesis selection should not be guided solely by the objective of minimizing PPM. Considerations such as valve durability, ease of coronary access, and the feasibility of future valve-in-valve interventions, especially in younger patients requiring lifelong valve management, may be equally or more important. As highlighted by the SMART and CHOICE trials, BEV were found to have higher rates of valve deterioration than SEV which, for younger patients in particular, is likely an important consideration.^3^^,^^13^^,^^25^ Alternatively, BEVs likely provide improved coronary access for patients at increased risk of coronary artery disease and facilitate future interventions. As we continue to better understand the concept of PPM in patients receiving TAVR, long-term outcomes, especially in low-risk patients, are important. Given the multiple assumptions by the echo equations, developed initially to assess native aortic valves, it may be time to modify how we assess and react to valve gradients. This may be prosthetic valve type-specific or perhaps a standardized invasive assessment at the time of deployment as a quality metric. If mortality does not become an issue with PPM in TAVR, quality of life should be assessed next to understand any negative impacts. Ultimately, a patient-specific approach integrating hemodynamic performance, durability, coronary access, and future intervention feasibility should guide prosthesis selection rather than a singular focus on PPM reduction.

### Limitations

This study is subject to several limitations, most notably due to its retrospective and observational nature. As data were sourced from a single regional health care system, the generalizability of the findings to a broader TAVR population, particularly to centers with varying levels of operator experience and from different geographical regions, may be limited. Imbalances in baseline characteristics between groups (male vs female and BEV vs SEV) introduces potential confounders that may not be fully addressed by our adjusted analyses. Additionally, the small sample sizes in the severe PPM subgroup also limit the statistical power for certain comparisons. Our reliance on both predicted and measured EOA thresholds is in keeping with current guidelines, but this may not fully capture the complexity of patient-prosthesis interactions. This may be further accentuated with PPM_P_ in particular as there may be device recoil following deployment.^15^ Echocardiographic findings were per site and not core lab adjudicated. Future research should focus on prospective validation of these findings, with an emphasis on sex-specific differences in PPM tolerance and ventricular remodeling. Additionally, further analysis including quality of life metrics should be considered, particularly in the absence of significant mortality differences.

## Conclusion

This study highlights the multifactorial relationship between sex, valve type, and PPM severity in shaping long-term outcomes following TAVR. While PPM disproportionately affects women, their survival outcomes were not negatively impacted. Although SEV offered improved hemodynamics, this did not translate into improved survival. Ultimately, these data underscore the importance of a patient-specific approach, encompassing prosthesis selection, anatomical considerations, as well as lifelong management considerations, in an effort to optimize outcomes in this diverse patient population.
