# Supplementary material for: Incidence and Sex-Based Comparison of Prosthesis-Patient Mismatch in Patients Undergoing Transcatheter Aortic Valve Replacement
Source: J Soc Cardiovasc Angiogr Interv. 2025 May 1;4(7):103621. doi: 10.1016/j.jscai.2025.103621 (PMC12418439; doi:10.1016/j.jscai.2025.103621)

**Supplementary Appendix**

**Supplementary Table 1.** Predicted EOA based on transcatheter valve type and size.

| **Parameters** | **All patients**  **(N=3016)** | **Predicted EOA (cm²)*** |
| --- | --- | --- |
| **Balloon-expandable devices** | 2260 (74.9%) |  |
| **Sapien** | 147 (4.87%) |  |
| 23 mm | 71 (2.35%) | 1.56 |
| 26 mm | 76 (2.52%) | 1.84 |
| **Sapien XT** | 136 (4.51%) |  |
| 23 mm | 40 (1.33%) | 1.41 |
| 26 mm | 67 (2.22%) | 1.74 |
| 29 mm | 29 (0.96%) | 2.06 |
| **Sapien 3** | 1977 (65.6%) |  |
| 20 mm | 45 (1.49%) | 1.22 |
| 23 mm | 622 (20.6%) | 1.45 |
| 26 mm | 849 (28.1%) | 1.74 |
| 29 mm | 461 (15.3%) | 1.89 |
| **Self-expandable devices** | 756 (25.1%) |  |
| **CoreValve** | 325 (10.8%) |  |
| 23 mm | 18 (0.60%) | 1.12 |
| 26 mm | 116 (3.85%) | 1.74 |
| 29 mm | 148 (4.91%) | 1.97 |
| 31 mm | 43 (1.43%) | 2.15 |
| **Evolut R/Pro** | 431 (14.28%) |  |
| 23 mm | 24 (0.79 %) | 1.09 |
| 26 mm | 138 (4.57 %) | 1.69 |
| 29 mm | 158 (5.23%) | 1.97 |
| 34 mm | 111 (3.68 %) | 2.60 |
| * According to Hahn et al.^16^ | | |

**Supplementary Figure 1.** Post-procedural prosthetic mean gradient, EOA and EOAi between male and female recipients.


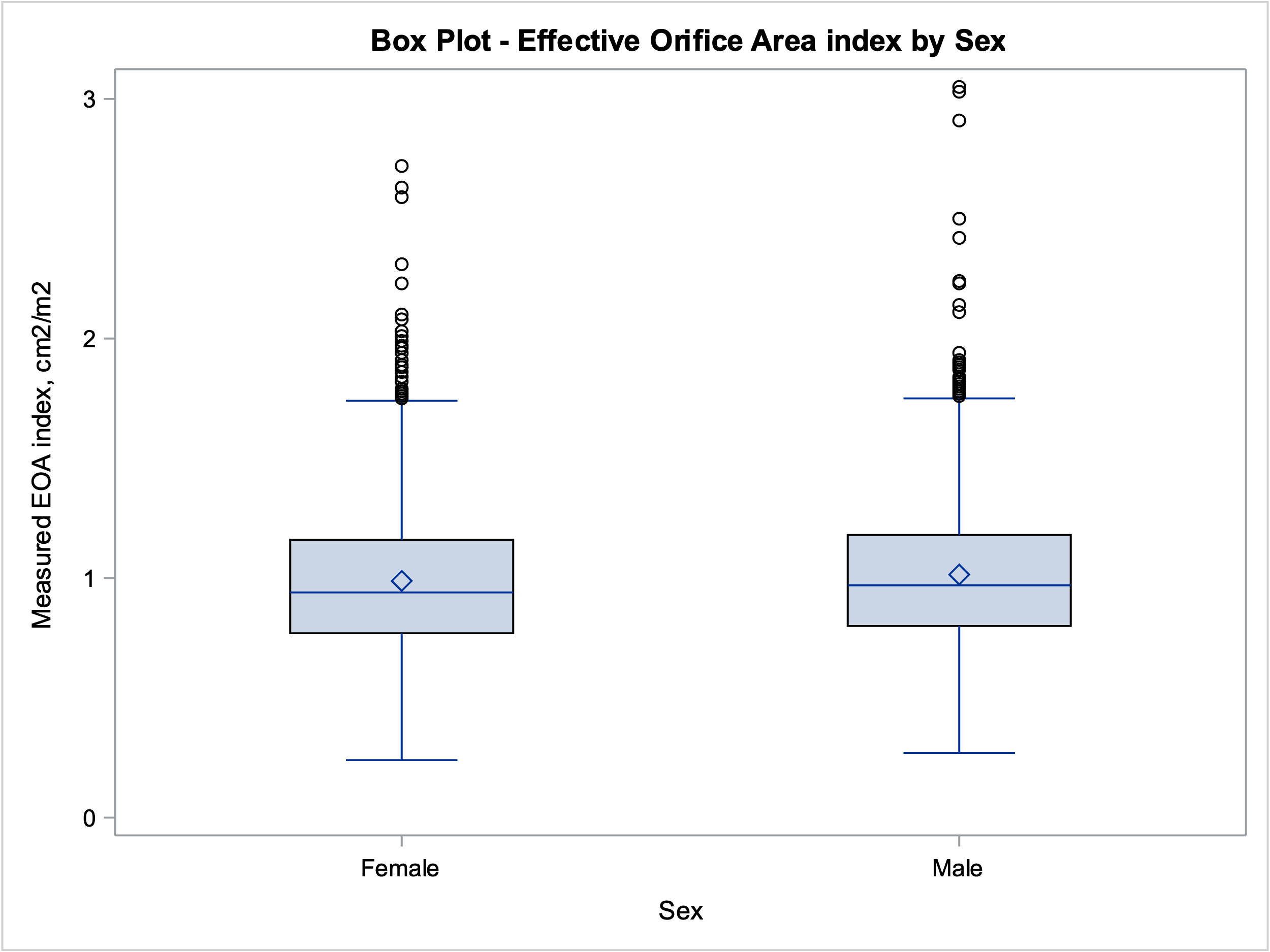

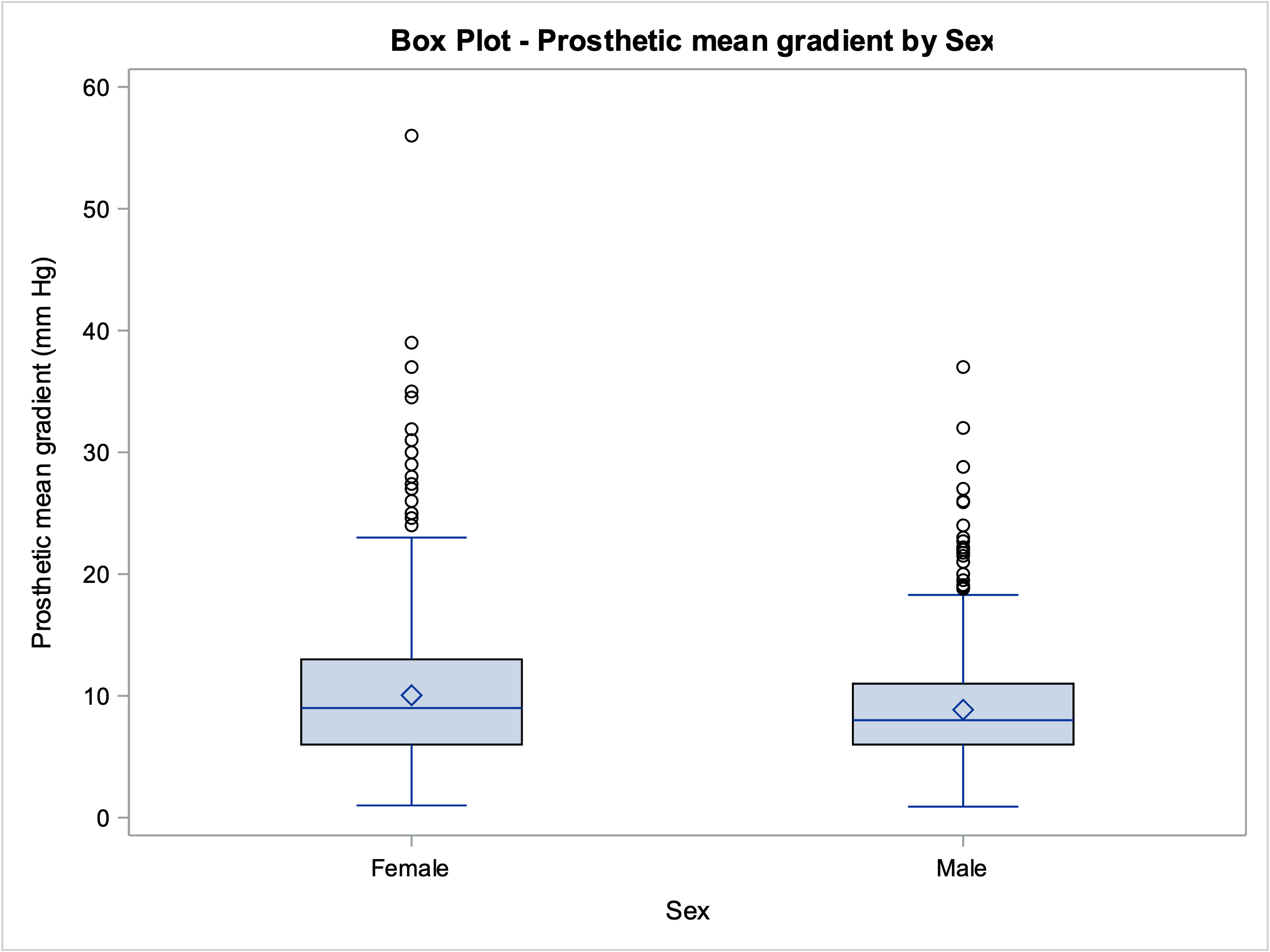

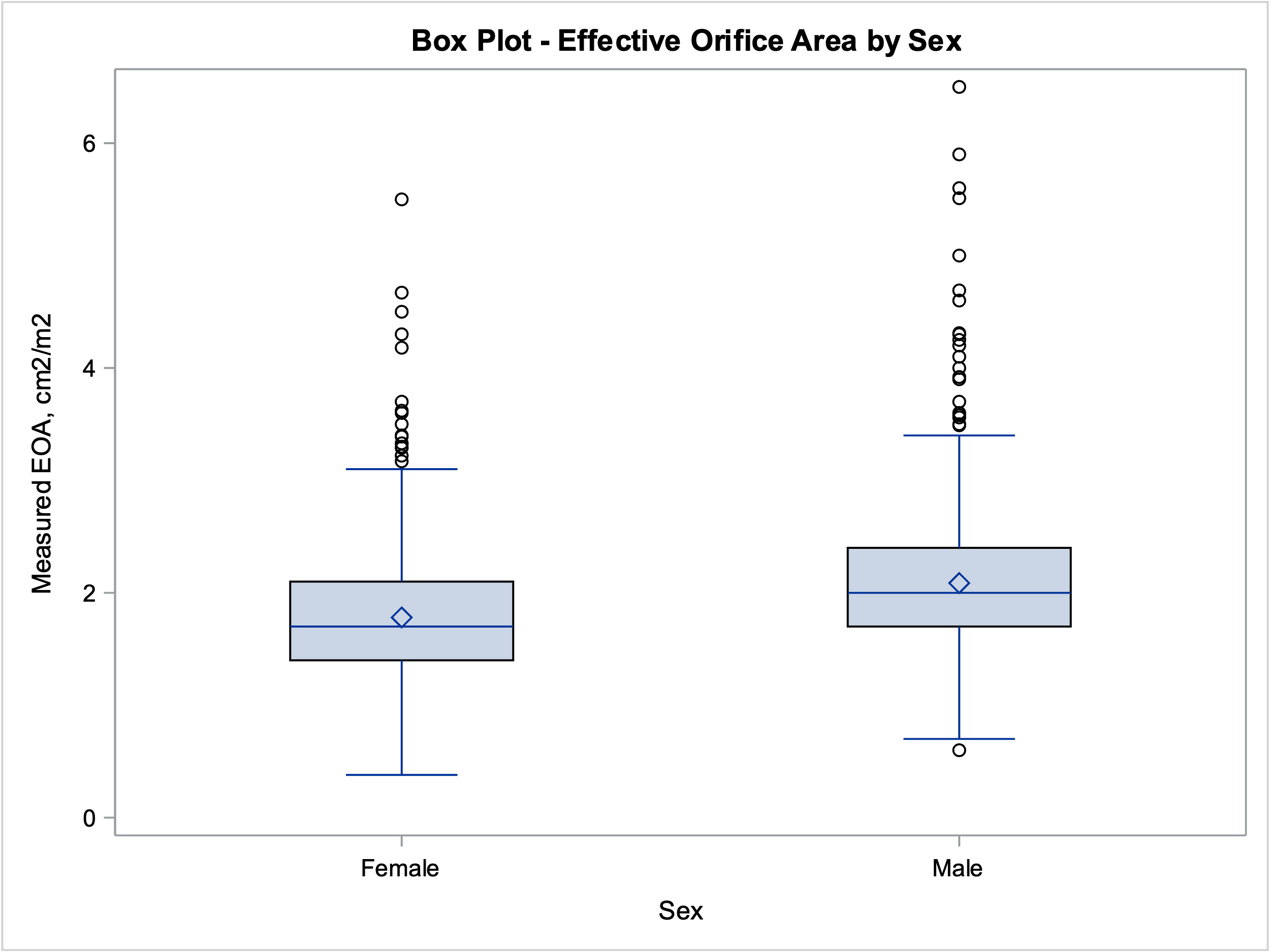


**Supplementary Figure 2.** Post-procedural prosthetic mean gradient, EOA and EOAi in female patients based on valve type (BEV vs SEV).


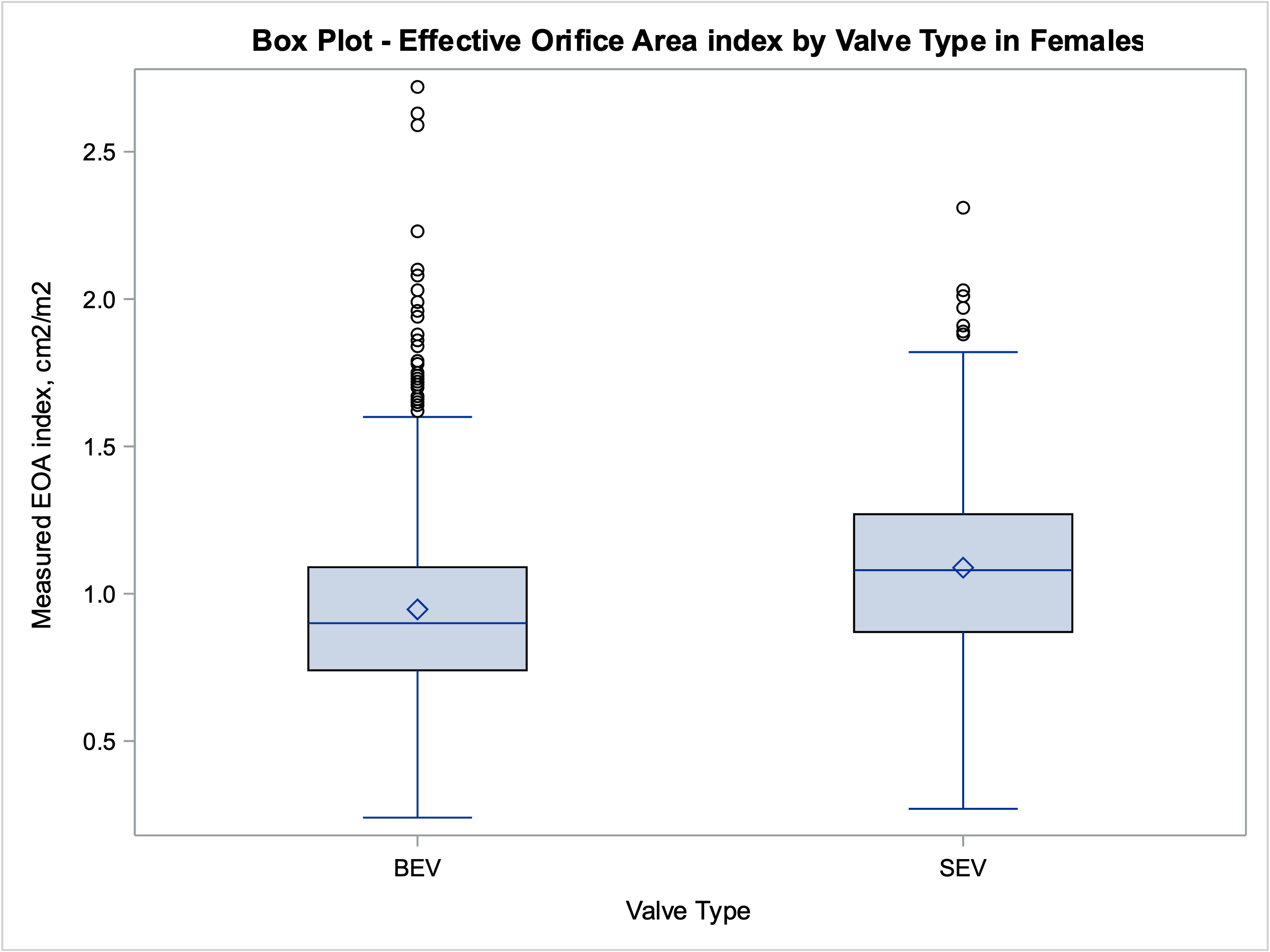

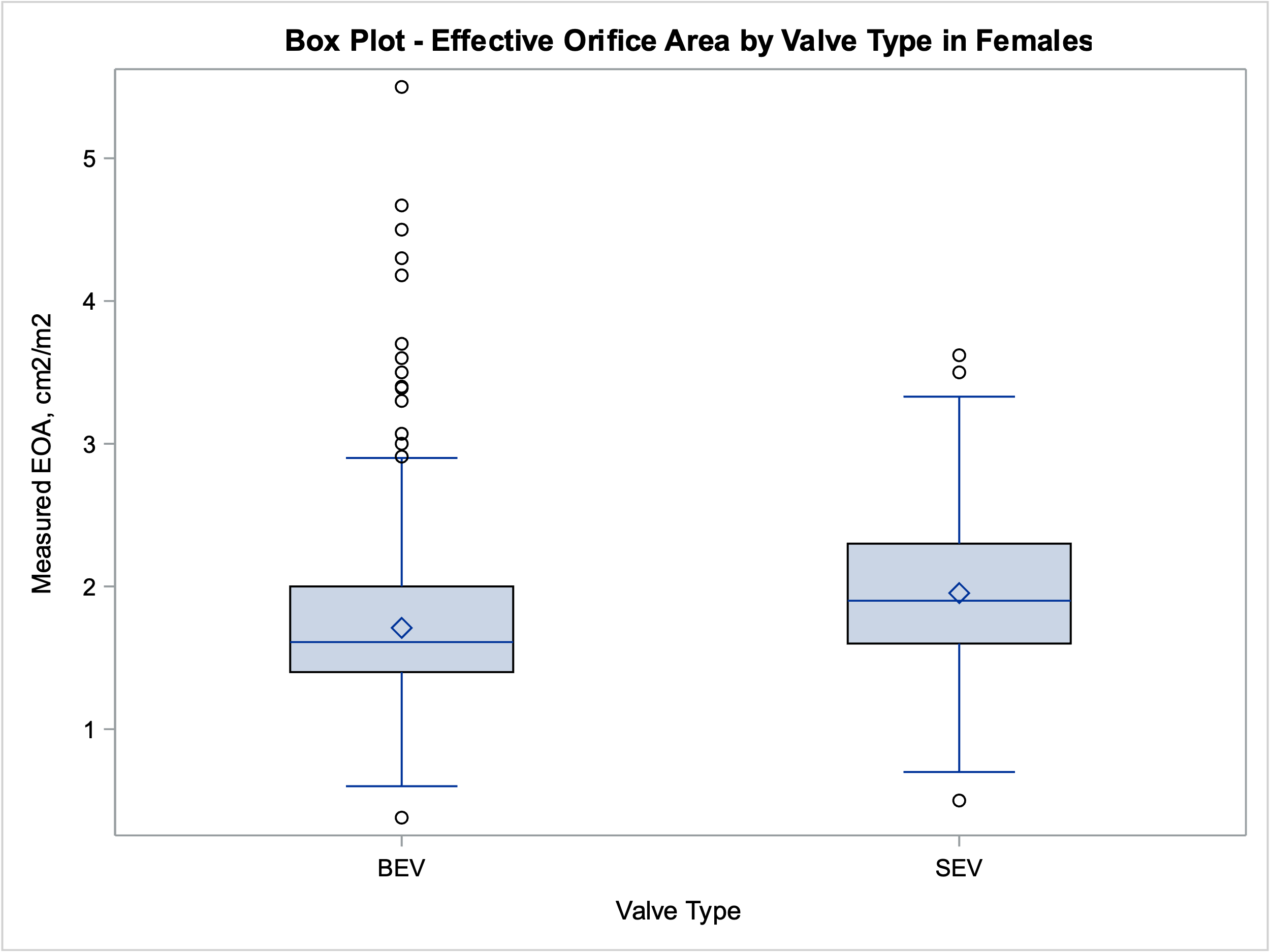

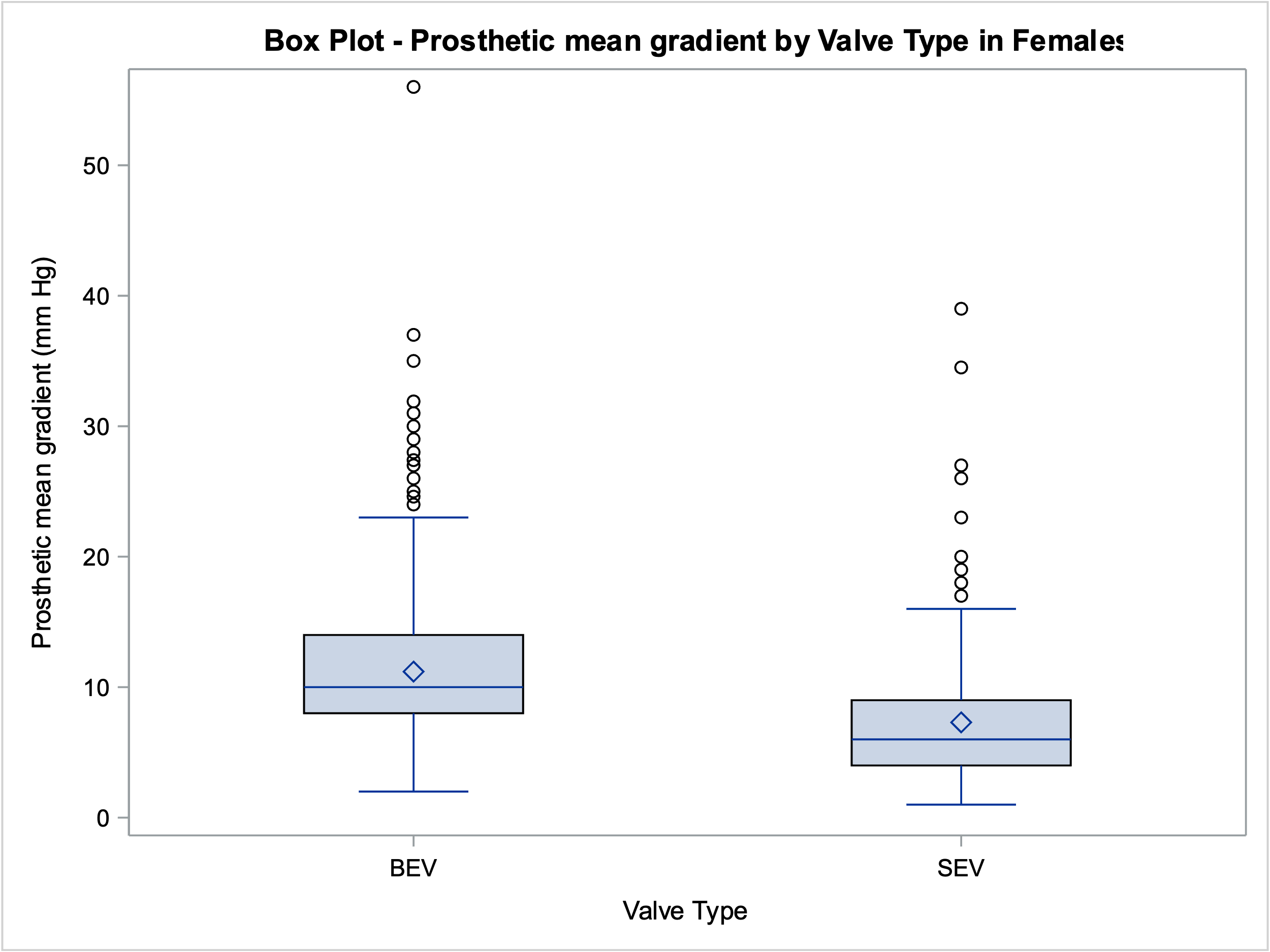


**Supplementary Figure 3.** Kaplan-Meyer 5-year survival analysis with adjusted hazard ratio’s comparing moderate and severe PPMp and PPMm to no PPM in male patients

Moderate vs. None

HR_adjusted_ 1.09, 95% CI (0.95,1.42); p=0.504

Severe vs. None

HR_adjusted_ 1.44, 95% CI (0.97,2.14); p=0.0695


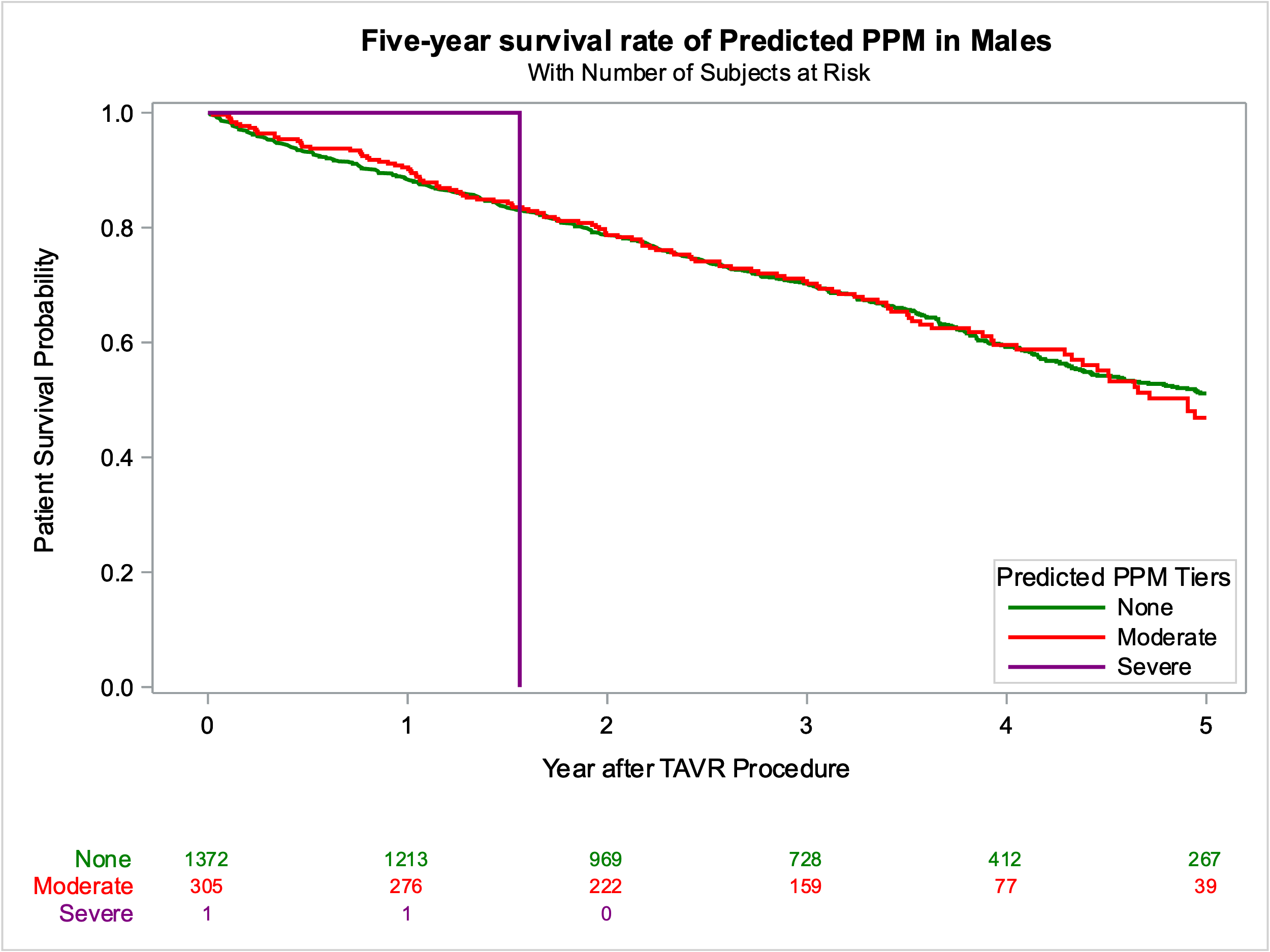


Moderate vs. None

HR_adjusted_ 1.51, 95% CI (1.11,2.06); p=**0.0096**

Severe vs. None

HR_adjusted_ 7.25, 95% CI (0.95,55.05); p=0.0557


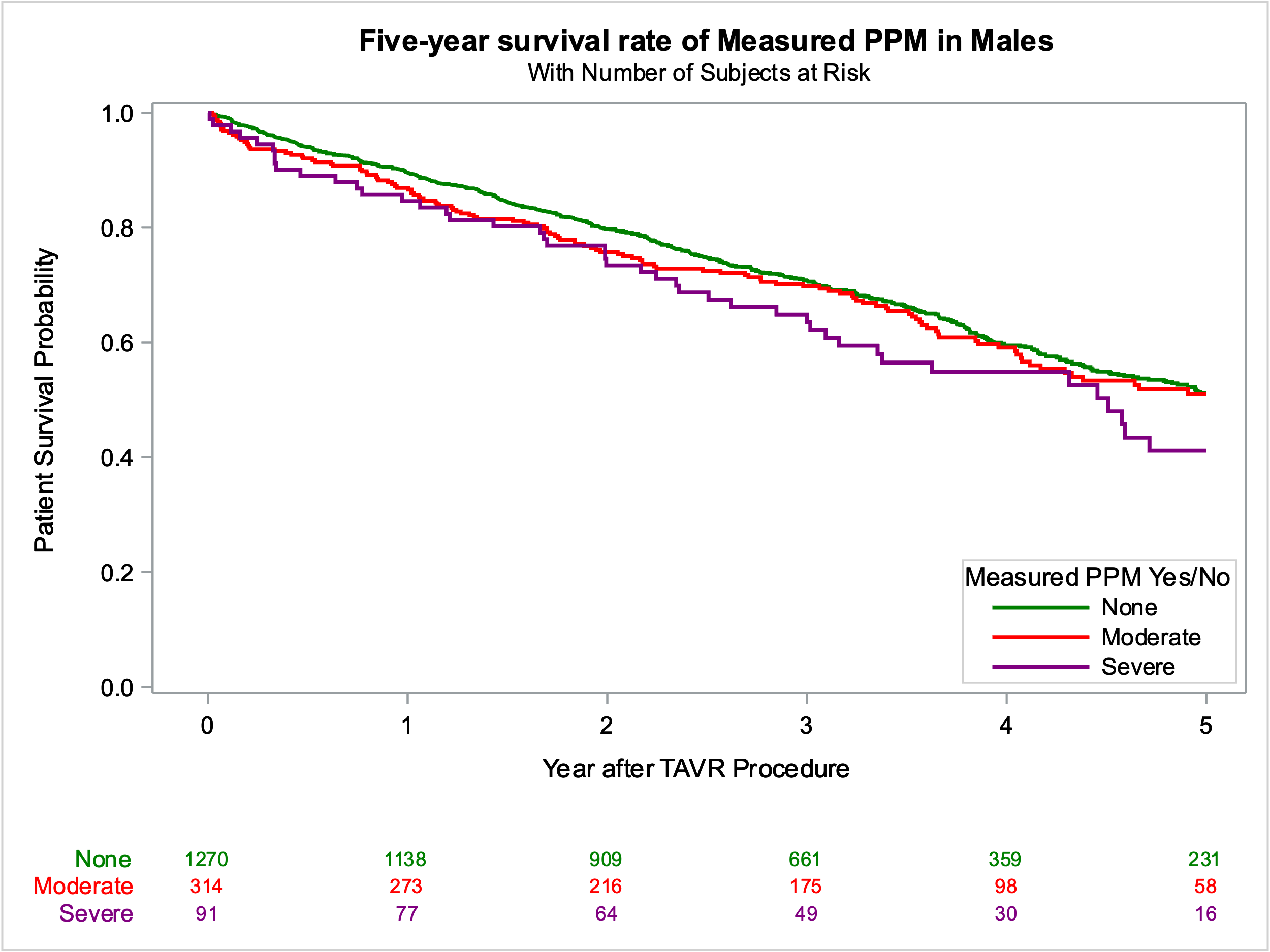


**Supplementary Figure 4.** Five-year Kaplan-Meier survival analysis stratified by valve type (BEV vs. SEV) and predicted or measured prosthesis-patient mismatch severity in female patients. A: Overall PPMp. B: Moderate PPMp. C: Severe PPMp. D: Overall PPMm. E: Moderate PPMm. F: Severe PPMm


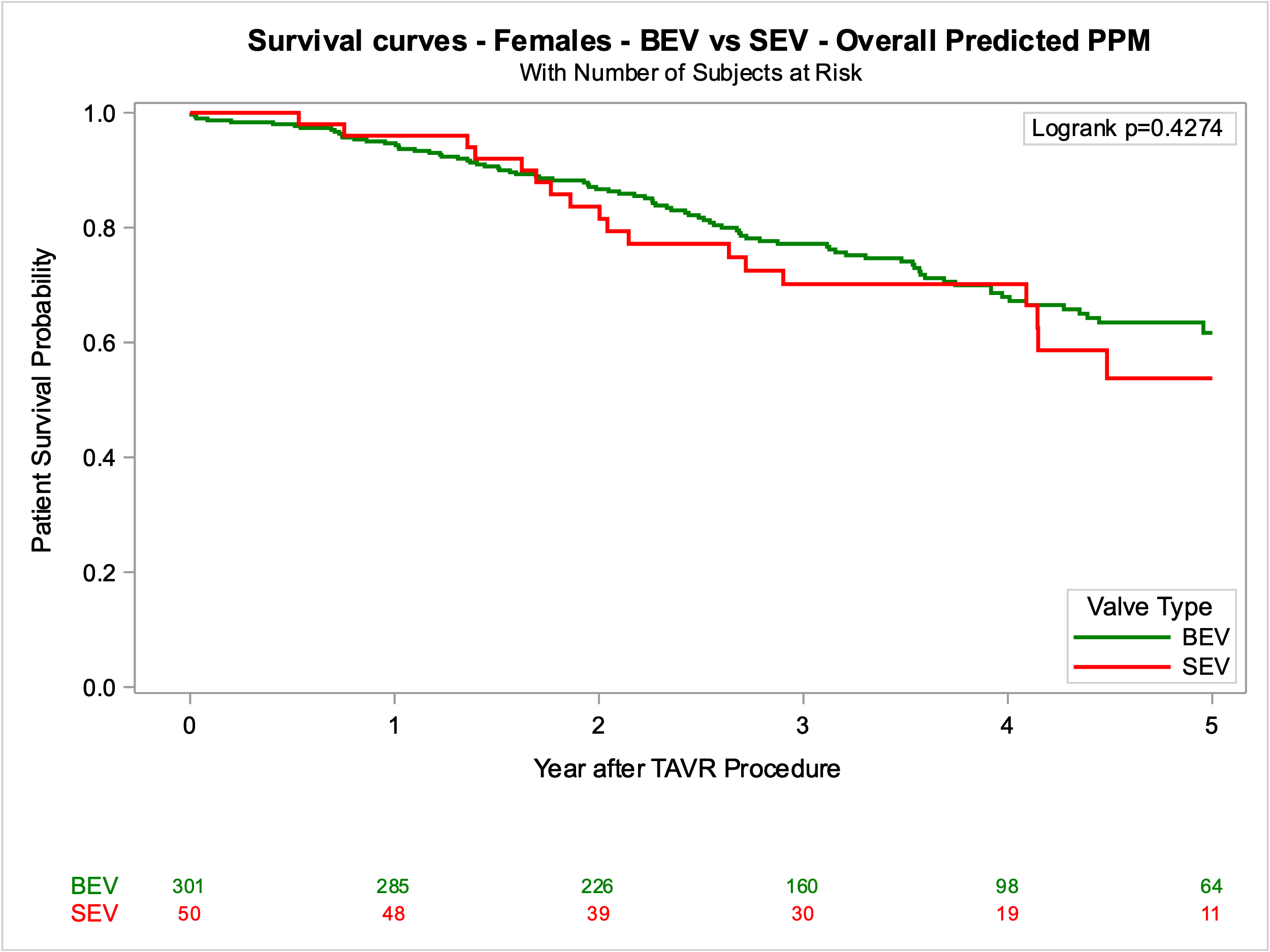

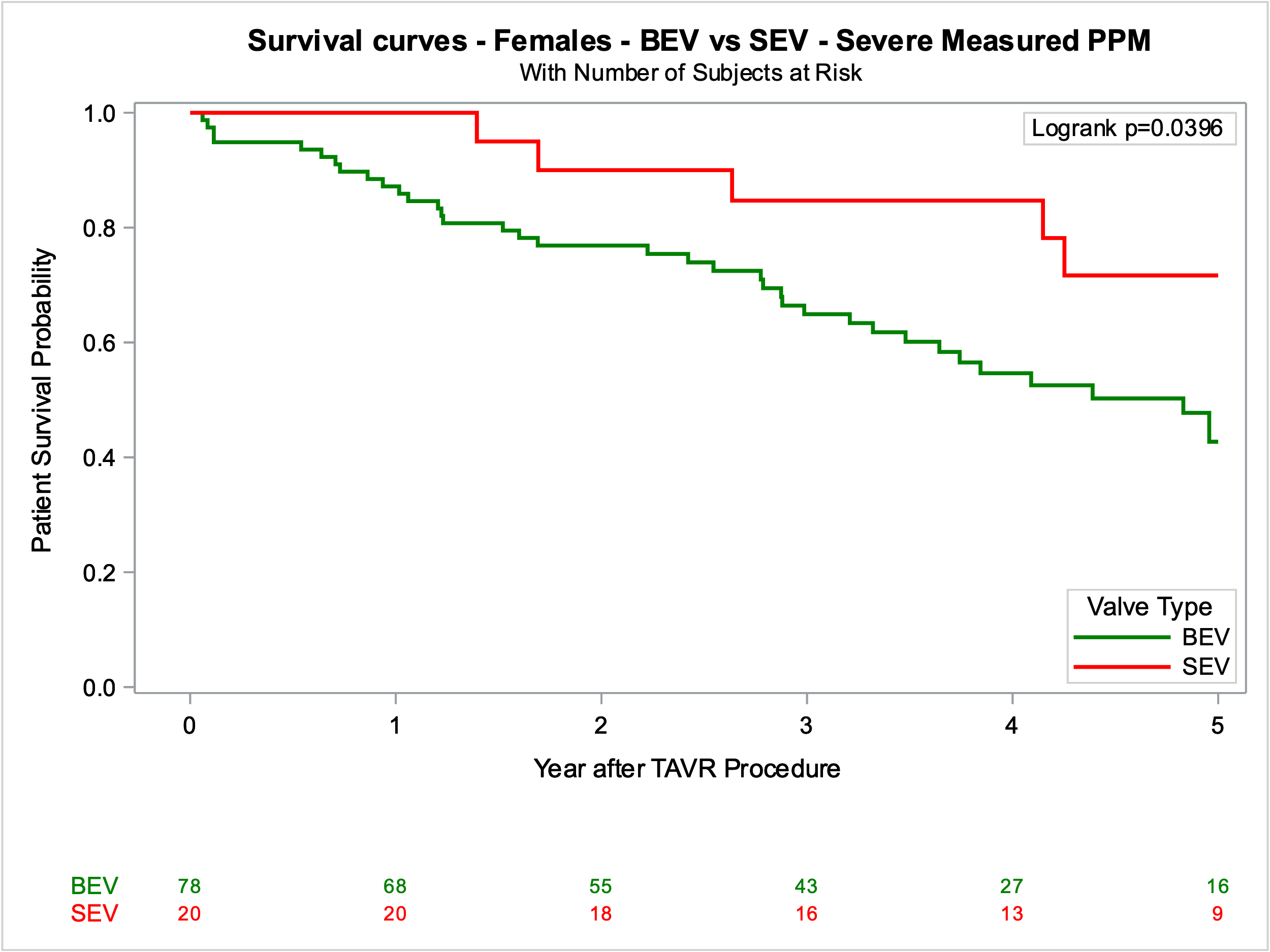

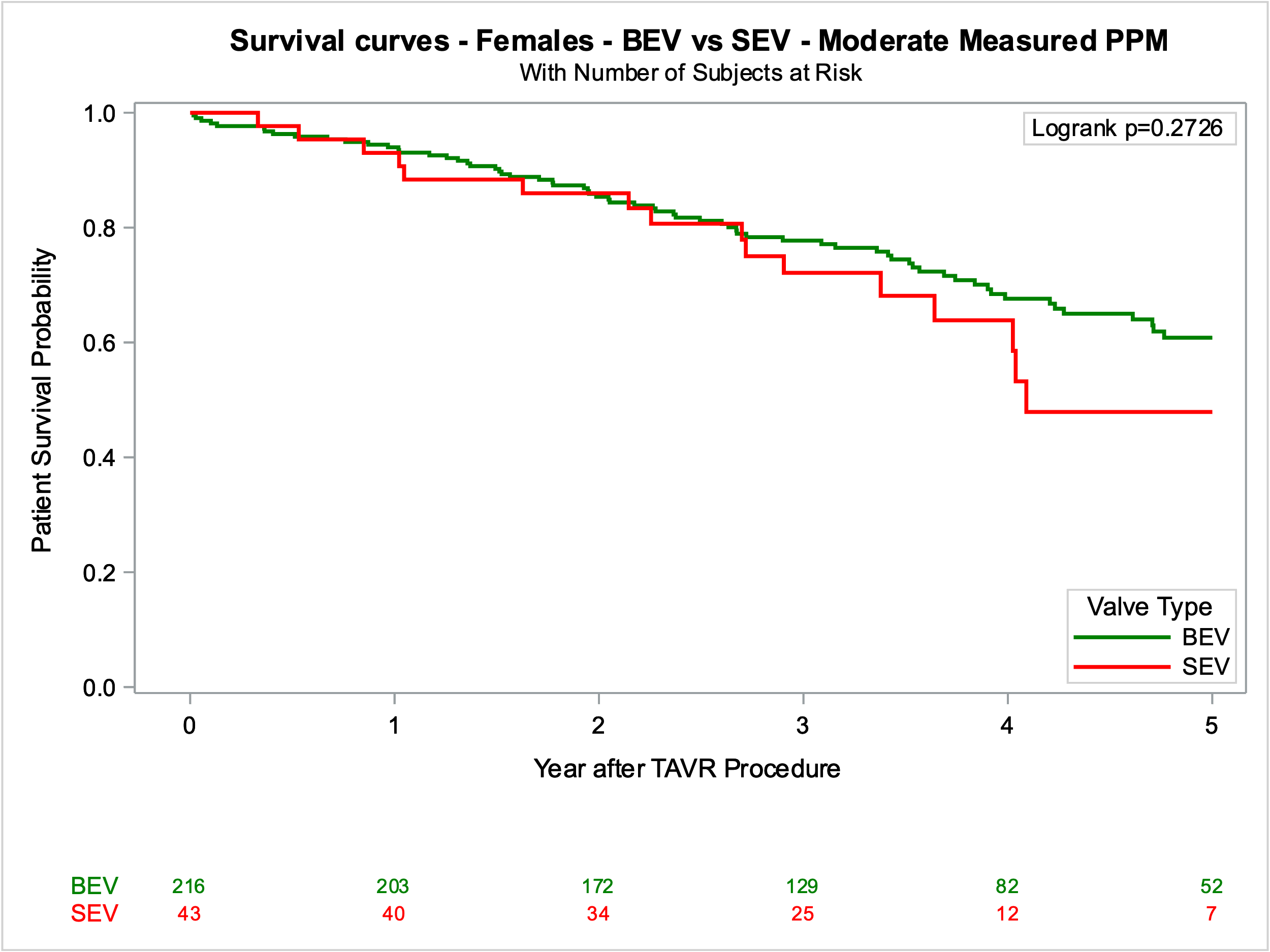

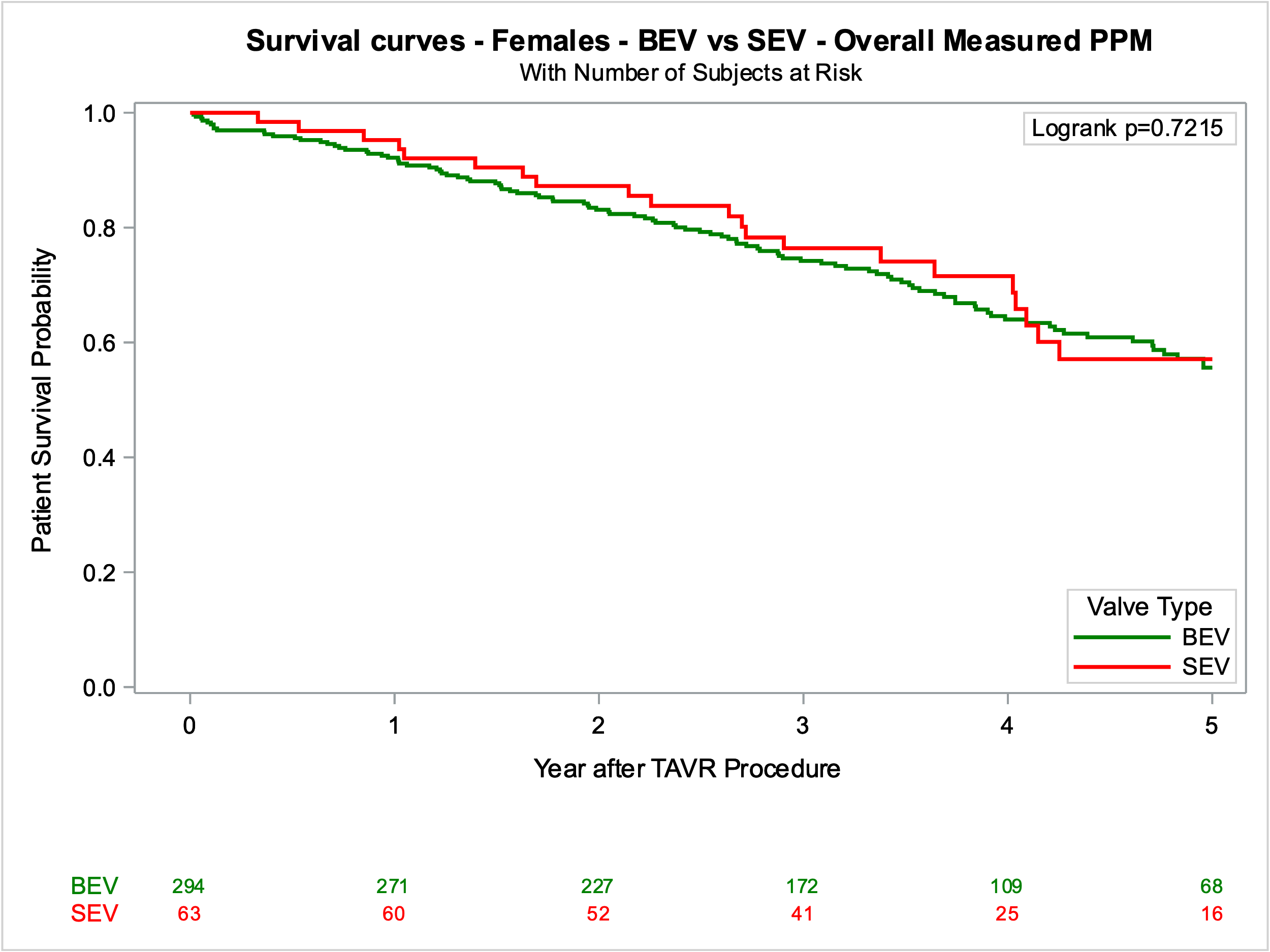

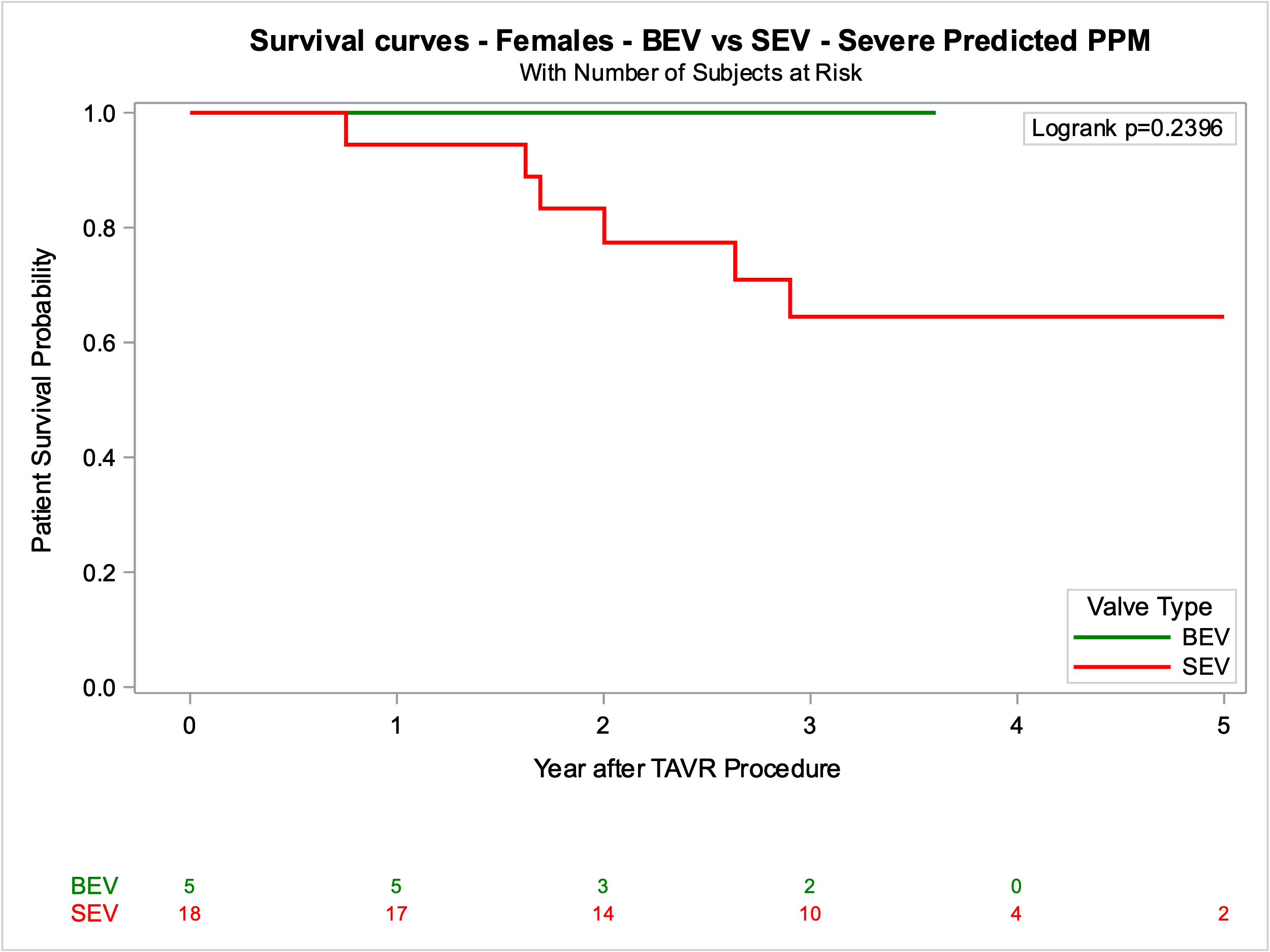

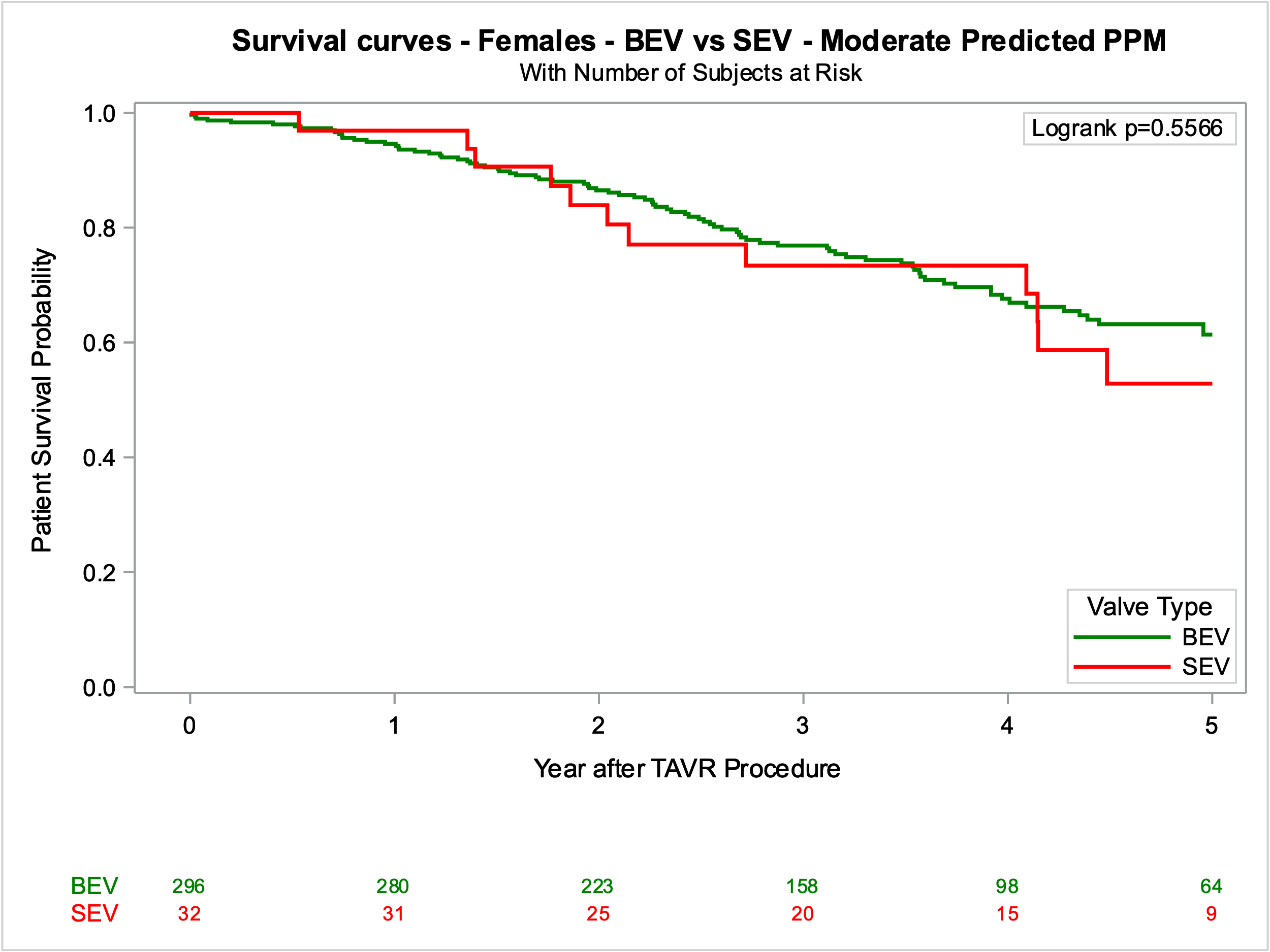

Supplement: Supplementary Material [file mmc1.docx]
